# Gastric duplication cyst masquerading as a mucinous pancreatic cyst: case report and literature review

**DOI:** 10.1308/003588414X13824511649977

**Published:** 2014-01

**Authors:** CE Bailey, MB Fritz, L Webb, NB Merchant, AA Parikh

**Affiliations:** Vanderbilt University Medical Center, Nashville, TN,US

**Keywords:** Mucinous cyst, Duplication, Pancreatic cyst, Carcinoembryonic antigen

## Abstract

Gastric duplication cysts are rare cystic neoplasms that are often difficult to distinguish from other entities. We describe a healthy 44-year-old woman who presented with acute right lower quadrant abdominal and flank pain as well as chronic nausea and constipation. Her physical examination was unremarkable but contrasted computed tomography revealed a 6cm cystic lesion between the stomach and body of the pancreas. Endoscopic ultrasonography and fluid analysis were consistent with a mucinous cyst with a markedly elevated fluid carcinoembryonic antigen level. The patient subsequently underwent a laparoscopic distal pancreatectomy, which was converted to an open procedure when the lesion was noted to be adherent to the coeliac axis. Intraoperative endoscopy revealed no abnormality. Final pathology revealed a gastric duplication cyst. The patient recovered well and was asymptomatic on follow-up. In this report, we discuss the incidence, natural history and management of this rare entity.

Gastric duplication cysts (GDCs) are rare and benign but difficult to distinguish from cystic neoplasms of the pancreas or pseudocysts on imaging. Endoscopic ultrasonography guided fine needle aspiration (EUS-FNA) and fluid analysis is the most common modality used in attempting to distinguish between these entities. Nevertheless, in some cases, a definitive diagnosis may not be possible preoperatively. Resection is usually performed in symptomatic patients while the choice to resect or observe in asymptomatic patients is less clear. In this report, we describe a patient who presented with a large cystic lesion that appeared to be a mucinous pancreatic neoplasm by preoperative imaging and fluid analysis. On surgical exploration, the diagnosis of a GDC was made and resection was performed. A review of the literature on the diagnosis and management of GDCs is also discussed.

## Case history

**Figure 1:**
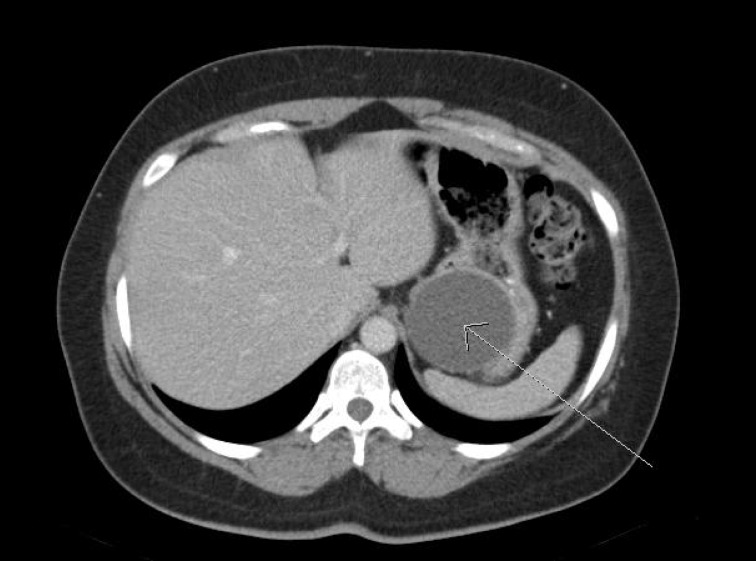
Contrast enhanced computed tomography of a gastric duplication cyst (arrow)

**Figure 2:**
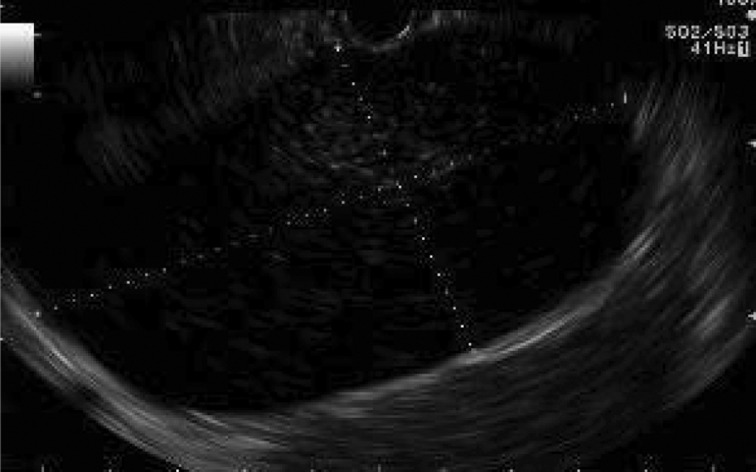
Endoscopic ultrasonography of a gastric duplication cyst

A 44-year-old woman presented with a 3-day history of right flank and lower quadrant abdominal pain associated with nausea and constipation. Her past medical, family and medication history were otherwise non-contributory and her physical examination was unremarkable. She underwent contrast enhanced computed tomography, demonstrating a 6cm cystic lesion between the stomach and body/tail of the pancreas (Fig 1). She subsequently underwent EUS-FNA, which revealed normal pancreatic echotexture and a cyst measuring 6cm × 9cm that was free of internal septations or associated masses (Fig 2) but compressed the stomach. FNA of the cyst demonstrated no evidence of malignancy but did show the presence of extracellular mucin as well as a carcinoembryonic antigen (CEA) level of 12,476.5ng/ml and a carbohydrate antigen (CA) 19-9 level of 6iu/ml, suggesting the diagnosis of a mucinous pancreatic cystic neoplasm. The patient was therefore referred for surgical resection.

A laparoscopic distal pancreatectomy was planned. At the time of the laparoscopic exploration, entrance to the lesser sac was first obtained and, on mobilising the posterior wall of the stomach, the lesion was noted to be tightly adherent to both the stomach and pancreas. The pancreas was mobilised free of the cyst and it was evident that the lesion originated from the stomach. Intraoperative endoscopy was performed and no mucosal abnormalities of the stomach were noted.

On further mobilisation, the cyst was adherent to the coeliac axis and the procedure was converted to an open resection. The stomach was fully mobilised and the gastric duplication cyst was noted to be arising from the posterior wall. It was dissected off the coeliac vessels and an *enbloc* resection of the cyst along with a portion of the posterior wall of the stomach with a surgical stapler was performed. Repeat oesophagogastroduodenoscopy revealed the presence of an intact staple line on the posterior stomach wall and no evidence of bleeding or leak. The gross margins were uninvolved.

**Figure 3:**
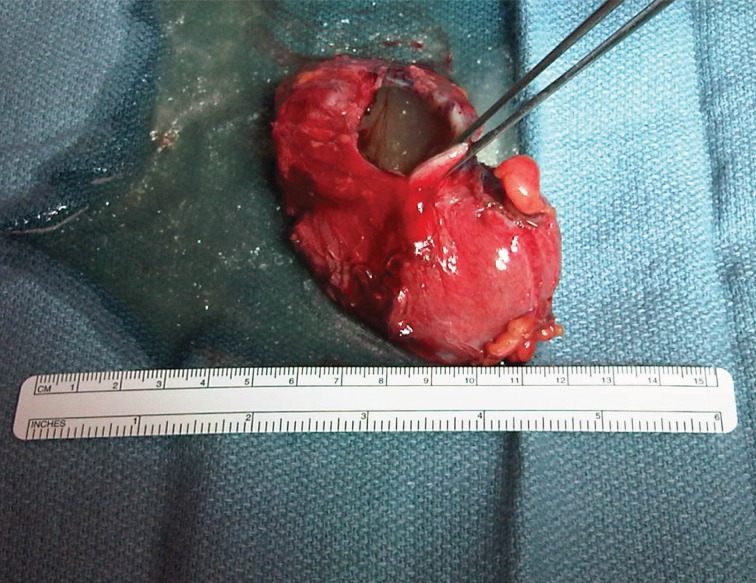
Pathological appearance of a gastric duplication cyst with associated mucinous fluid

**Figure 4:**
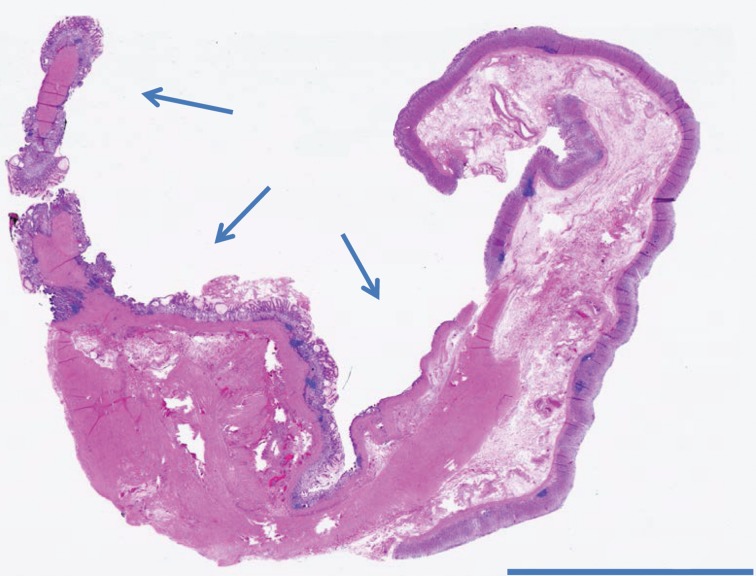
Haematoxylin and eosin stained section of gastric duplication cyst (low power)

**Figure 5:**
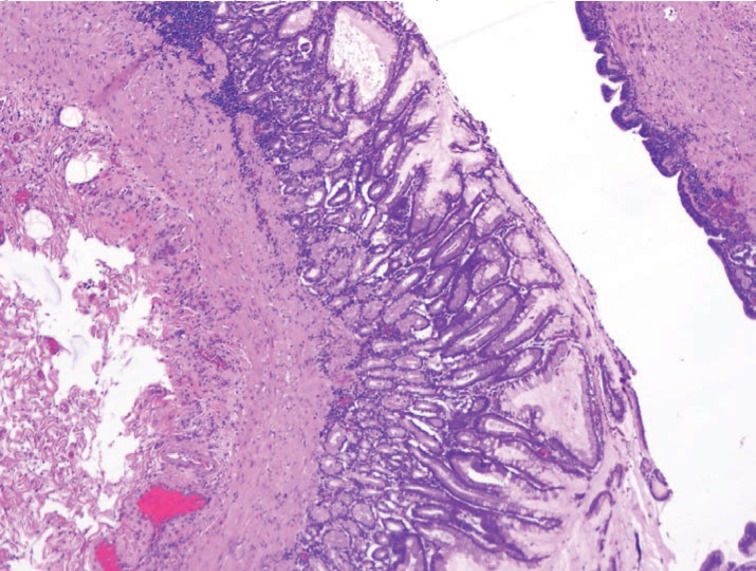
Haematoxylin and eosin stained section showing gastric antral-like epithelium lining the gastric duplication cyst (higher power)

Final pathology revealed a 9.5cm × 4.5cm × 2.0cm cyst with a smooth internal cyst wall consistent with a GDC (Figs 3–5). The patient was discharged home on postoperative day 4, doing well and tolerating a regular diet without any complications. On follow-up, she had complete resolution of her pain and nausea, and a return to regular bowel function and activity.

## Discussion

GDCs occur in approximately 0.001% of the general population, and represent 2–8% of all alimentary tract duplication cysts behind those of the ileum, oesophagus and colon.^1,2^ They are most commonly present on the distal greater curve of the stomach and, in general, do not directly communicate with the lumen of the alimentary tract. Theories for the development of duplication cysts include that they represent transient enteric diverticula that fail to degenerate, that the failure of the separation of notochordal plates results in traction diverticula that develop into duplications and that embryonic enteric longitudinal folds fuse with a bridge of tissue in between, resulting in one tract developing into the true alimentary tract and the second into the duplication.^3^

In the paediatric population, patients often present with an abdominal mass, gastrointestinal obstruction or anaemia^4^ while adults usually present with abdominal pain, bloating or nausea.^5^ Other non-specific complaints such as vomiting, weight loss, haematemesis and melaena may also be present although some patients may be completely asymptomatic.^4,5^ Associated congenital anomalies can include oesophageal and duodenal diverticula, other duplication cysts in the alimentary tract, annular and ectopic pancreas, and spinal anomalies such as hemivertebrae and spina bifida. Complications of duplication include torsion of pedunculated cysts, malignant changes into carcinoma, infection, peptic ulcer, obstruction, haemorrhage, pancreatitis and fistula formation.

The differential diagnoses of a suspected GDC often include cystic pancreatic neoplasms and pseudocysts. With the development of more modern imaging techniques including EUS with fluid analysis, the diagnosis is now more often made before surgery. Although GDCs typically have elevated fluid CA19-9 levels without elevated CEA, our patient had a markedly elevated fluid CEA level but a normal fluid CA19-9 level as well as the presence of extracellular mucin. This is more consistent with a mucinous cystic neoplasm of the pancreas. Elevated fluid CEA levels in pancreatic cysts are not always indicative of mucinous cysts. However, large cysts with CEA levels over 6,000ng/ml combined with the presence of mucin are worrisome for mucinous cysts with high malignant potential and are almost always resected uniformly.

Once diagnosed, the treatment of most foregut duplication cysts is surgical excision due to the majority being symptomatic and/or complicated at the time of presentation as well as reports of the development of malignancies within them.^2^ GDCs should ideally be treated by complete surgical excision without violation of the gastric lumen or by segmental gastric resection. If large, partial excision of the duplication along with mucosal stripping of the common wall or internal drainage into the stomach can be helpful as opposed to a formal gastrectomy.

## Conclusions

This report demonstrates the difficulty in distinguishing between a GDC and a cystic mucinous pancreatic neoplasm, even with modern imaging and diagnostic modalities such as EUS. In the case of our patient, her GDC was symptomatic and, consequently, even if the correct diagnosis had been made, resection would have been recommended. In asymptomatic patients, however, indications for resection as well as the operative approach may be different. Surgeons should therefore be aware of its possibility.

## References

[1] Coit DG, Mies C. Adenocarcinoma arising within a gastric duplication cyst. *J Surg Oncol* 1992; : 274–277. 10.1002/jso.2930500417 1640716

[2] McLetchie NG, Purves JK, Saunders RL. The genesis of gastric and certain intestinal diverticula and enterogenous cysts. *Surg Gynecol Obstet* 1954; : 135–141. 13187191

[3] O’Donnell PL, Morrow JB, Fitzgerald TL. Adult gastric duplication cysts: a case report and review of literature. *Am Surg* 2005; : 522–525. 16044936

[4] D’Journo XB, Moutardier V, Turrini O *et al.* Gastric duplication in an adult mimicking mucinous cystadenoma of the pancreas. *J Clin Pathol* 2004; : 1,215–1,218. 15509688 10.1136/jcp.2004.019091 PMC1770488

[5] Azzie G, Beasley S. Diagnosis and treatment of foregut duplications. *Semin Pediatr Surg* 2003; : 46–54. 12520472 10.1053/spsu.2003.50000

